# Damage Burden in Polish Patients with Antiphospholipid Syndrome Measured Using Damage Index for Antiphospholipid Syndrome (DIAPS)

**DOI:** 10.3390/biomedicines13071671

**Published:** 2025-07-08

**Authors:** Ewa Haladyj, Barbara Stypinska, Agata Matusiewicz, Wojciech Kunisz, Marzena Olesinska, Agnieszka Paradowska-Gorycka

**Affiliations:** 1 Eli Lilly and Company, Indianapolis, IN 46285, USA; 2 Department of Molecular Biology, National Institute of Geriatrics, Rheumatology and Rehabilitation, 02-637 Warsaw, Poland; 3 Department of Connective Tissue Diseases, National Institute of Geriatrics, Rheumatology and Rehabilitation, 02-637 Warsaw, Poland; 4 Department of Radiology, National Institute of Geriatrics, Rheumatology and Rehabilitation, 02-637 Warsaw, Poland

**Keywords:** antiphospholipid syndrome, antiphospholipid antibodies, DIAPS, SLE

## Abstract

**Objectives**: We aimed to quantify the damage burden measured using the Damage Index for Antiphospholipid Syndrome (DIAPS) in patients with antiphospholipid syndrome (APS) and identify patients with high damage as well as any correlations of damage with subclinical atherosclerosis. **Methods**: Patient damage was assessed via DIAPS. Based on demographic, clinical and laboratory characteristics, patients were divided into two subgroups: thrombotic APS patients with high vs. low damage, and non-thrombotic aPL-positive patients with vs. without damage. Participants underwent carotid/femoral ultrasound for atherosclerotic plaque detection and carotid–femoral and carotid-radial pulse wave velocity (PWV). **Results**: We included 112 patients with an APS diagnosis, 57 (50.9%) with primary APS and 55 (49.1%) with associated SLE. Cardiovascular (CVD) risk factors and complications were significantly more frequent in the thrombotic group, as well as in patients with high damage within the thrombotic group. We did not identify any risk factors for increased damage in the non-thrombotic group. Atherosclerotic plaque presence was present in 27 (24%) of the patients in this study with the same frequency in the APS and APS/SLE groups (*p* = 0.5446). Pulse wave velocity (PWV) was elevated in 27–32% patients according to analyzed arteries. Elevated PWV was more frequent in the APS group in comparison to APS/SLE only between carotid and radial arteries (*p* = 0.0012). Both atherosclerotic plaque presence and PWV did not correlate with damage severity. **Conclusions**: DIAPS indicates substantial damage in APS patients in our study. High organ damage mainly affected thrombotic patients and was related to CVD complications. At the same time, screening of subclinical atherosclerosis seems not to predict higher damage in APS patients.

## 1. Introduction

Antiphospholipid syndrome (APS) is currently described as a systemic, autoimmune disease revealing arterial, venous, or microvascular thrombosis, pregnancy morbidity, or non-thrombotic manifestations in patients with persistent antiphospholipid antibodies (aPL) [1]. Although APS may develop in patients with or without other autoimmune diseases, it most frequently coexists with systemic lupus erythematosus (SLE) [2].

Disease activity measures in SLE assess disease activity in individual organs or systems, while damage indices assess cumulative damage, which is important for predicting morbidity and mortality [3]. Organ damage measurement is well defined in SLE management, based on the Systemic Lupus International Collaborating Clinics/American College of Rheumatology (SLICC/ACR) Damage Index (SDI) [4]. Damage in APS patients progresses mostly due to recurrent thrombosis [5], which occurs frequently, with rates varying from 3.5% annually to 65% over longer periods [6,7]. At the same time, APS is a major predictor of irreversible organ damage and death in patients with systemic lupus erythematosus (SLE) [8].

The Damage Index for APS (DIAPS) for assessing damage accrual in thrombotic APS patients was developed and initially validated in Latin American patients [9]. DIAPS consists of 37 items: 22 from SDI and 15 newly added, scored up to 2 points (range 0–74 points) [9]. In the present work, we analyze DIAPS in patients with primary APS or coexisting with SLE, with or without thrombotic manifestations, to confirm or identify additional factors connected with high damage in the Polish population.

An important reason to measure damage due to APS is its direct connection to subclinical atherosclerosis. Recently, it was proven that patients with APS or aPLs have an increased risk of subclinical atherosclerosis and require early and disease-specific prevention of atherosclerosis [10].

## 2. Material and Methods

The research was conducted on a group of patients hospitalized in the years 2009–2015 at the Clinic and Polyclinic of Systemic Connective Tissue Diseases of the NIGRIR in Warsaw. The study included 112 patients with antiphospholipid syndrome diagnosed according to the Sydney consensus criteria (2006) [10]. Based on the presence of a concomitant disease (SLE, diagnosed based on the classification criteria according to SLICC (2012)), patients were divided into groups with primary APS only (APS) and with concomitant SLE (APS/SLE) [11]. Data from the history and physical examination as well as demographic data were collected in the patient’s examination protocol and then transferred to a computer database.

Based on medical history, physical examination and additional tests resulting from the specific clinical picture of APS and SLE and the involvement of internal organs observed in individual patients SLEDAI, DIAPS and SLICC were calculated. All patients underwent the following analytical tests at the Central Clinical Laboratory of NIGRiR, in accordance with the procedures recommended by the manufacturers: peripheral blood count (XT 2000 I analyzer, CBC-6DIF, Sys Mex, Kobe, Japan); biochemical tests: concentration of C-reactive protein (CRP), glucose, urea, creatinine, lipid profile, alanine transaminase (ALT) and aspartate transaminase (AST), creatine kinase (CK), lactate dehydrogenase (LDH) (Vitros 350 thin film tech, Ortho Clinical Diagnostics Jonson & Jonson Company, Warsaw, Poland); proteinogram (Capilaris Sebia analyzer, Horiba ABX, Warsaw, Poland); general urine test (URISYS analyzer, Roche Diagnostics, Warsaw, Poland); Biernacki’s test (ESR) (Sedi System RBC sedimentation analyzer), (ref. range ≤ 12 mm/1 h); high-sensitivity CRP (hsCRP), C3 and C4 (nephelometric IMMAGE 800); complement components; lupus anticoagulant (LAC) (screening and a confirmation test using excess phospholipids in the hexagonal phase and Russell’s viper venom, using the TOP 300 analyzer, InsSLEmentation Laboratory, Milano, Italy); anticardiolipin antibodies (aCL) IgM and IgG (ELISA according to the modified Gharavi method and our own modification) [11], antibodies against β2 glycoprotein I IgM, IgG, IgA, D1; aCL IgA (chemiluminescence ELISA QuantaLite B2 GPI IgM IgG BioFlash, Barcelona, Spain), antinuclear antibodies (indirect immunofluorescence test using Hep-2 cells); panel of autoantibodies for the following antigens: U1-RNP-70, U1-RNP-A, U1-RNP-C, Sm-B, Sm-D, Ro/SSA 60 kD, Ro/SSA 52 kD, La/SSB, RibP, PCNA, CENPB, Scl-70, Jo-1, dsDNA, histones (DOT-blot method Mikrogen, Barcelona, Spain); antibodies antiphosphatodyloserine IgM, IgG, IgA and antiprothrombin IgG, IgM (ELISA Inova Diagnostics BioFlash, Barcelona, Spain).

Each participant underwent ultrasound of carotid arteries with intima-media complex (IMT) measurement, as well as pulse wave velocity (PWV) between carotid, radial and femoral arteries. Examination was performed after 5 min of rest, on both carotid arteries including the bulb in 3 projections. Presence of atherosclerotic plaque (AP) was defined as IMT ≥ 1.3 mm. All examinations were conducted by the same ultrasonographist.

### 2.1. DIAPS Calculation

Calculation of DIAPS was performed as previously published by Amigo et al. [9]. As DIAPS was initially validated only for thrombotic APS, we divided aPL-positive patients into two groups and performed different analyses to understand the contribution of different clinical and laboratory characteristics in damage burden for each scenario: (i) a thrombotic group, and (ii) a non-thrombotic group (including obstetric APS) (similarly to other authors [12]).

To better describe patients with DIAPS, patients were divided into thrombosis and non-thrombotic groups based on the history of thrombosis. Patients from the thrombotic group were additionally divided into two groups according to high damage (DIAPS > 3) vs. low damage (DIAPS < 3) based on recent papers published by Medina et al. [11]. Since DIAPS was not initially validated for use in non-thrombotic patients, similarly to other authors [12], we analyzed only presence (DIAPS > 0) or absence of damage (DIAPS = 0). Groups were then compared regarding demographics, clinical and laboratory characteristics to identify variables associated with the high damage or presence of damage.

#### Statistical Analysis

Associations between categorical variables were evaluated using the chi-squared test or the Monte Carlo method with 2000 replicates when assumptions were not met. Continuous variables were compared using the *t*-test or the Mann–Whitney U test, depending on the distribution of the data. Mean values and standard deviations were reported for normally distributed variables, while the media along with the first and third quartiles were presented for variables with a skewed distribution. Pearson correlation analysis was performed to assess relationships between continuous variables. Differences were considered statistically significant at *p* < 0.05. No correction for multiple comparisons was applied. All calculations, tables, and figures were generated using the R statistical software (R Core Team, 2021).

## 3. Results

We included 112 patients with APS diagnosis, 57 (50.9%) with primary APS and 55 (49.1%) with associated SLE, both groups dominated by women. The median age was 48 years. The thrombotic group was larger (92.82%), with 20 patients (21.7%) with high damage. The non-thrombotic group mainly consisted of patients with damage (13.65%). Patients’ characteristics are summarized in Table 1.

Obstetric APS dominated in non-thrombotic patients. Cardiovascular (CVD) risk factors and complications were significantly more frequent in the thrombotic group, as well as in patients with high damage within the thrombotic group. In patients with DIAPS > 3, MI and reocclusion after PCI/CABG were significantly more frequent as well as the use of ASA.

There was no difference between patients with or without SLE when analyzed as thrombotic and non-thrombotic as well as with high/low damage (Table 2).

Damage measured via DIAPS was present in 86.6% of patients overall, with high damage in 17.86%. The median DIAPS was 2 (IQR 1–3, min = 0, max = 7) for all patients, 2 in the thrombotic group (IQR 1–3, min = 0, max = 7), and 1 in the non-thrombotic group (IQR 0–2, min = 0, max = 2), and the difference was statistically significant (*p* < 0.001). In the non-thrombotic group, 7 (35%) patients reported no damage (DIAPS > 0), and 20 (21.7%) in the thrombotic group had high damage (DIAPS ≥ 3) (Table 2). Damage measured via DIAPS was significantly higher in APS patients with thrombotic history (W = 499.5, *p*-value = 0.0009963), and it was independent of the presence of concomitant SLE diagnosis. DIAPS among non-thrombotic APS patients was low, not exceeding 3 points. In thrombotic patients, DIAPS above 3 was observed up to 20% (Table 2).

### Factors Associated with Increased Damage

Non-thrombotic patients had more frequent obstetric manifestations (15 (75%) vs. 24 (26%), *p* < 0.001), while thrombotic patients had significantly more frequent cardiovascular complications (45 (48%) vs. 0, *p* < 0.001) with more present as strokes (23 (25%) vs. 0, *p* = 0.052) and TIA (18 (19%) vs. 0, *p* = 0.044) but not myocardial infarction (*p* = 0.196) (Table 1).

In the thrombotic group, patients with high damage (DIAPS > 3) were more likely to have a history of cardiovascular complications (30 (41.6%) vs. 15 (75%), *p* = 0.017), myocardial infarction (4 (5.56%) vs. 6 (30.00%), *p* = 0.007) and reocclusion after PCI/CABG (0 vs. 3 (15%), *p* = 0.011) as well as associated with ASA treatment (16 (22.2%) vs. 13 (66%), *p* < 0.001) (Table 1). We did not identify any risk factors for increased damage in the non-thrombotic group.

In patients with concomitant SLE, median SLICC was higher in the thrombotic group; however, this was not statistically significant (Table 3).

**Figure 1:**
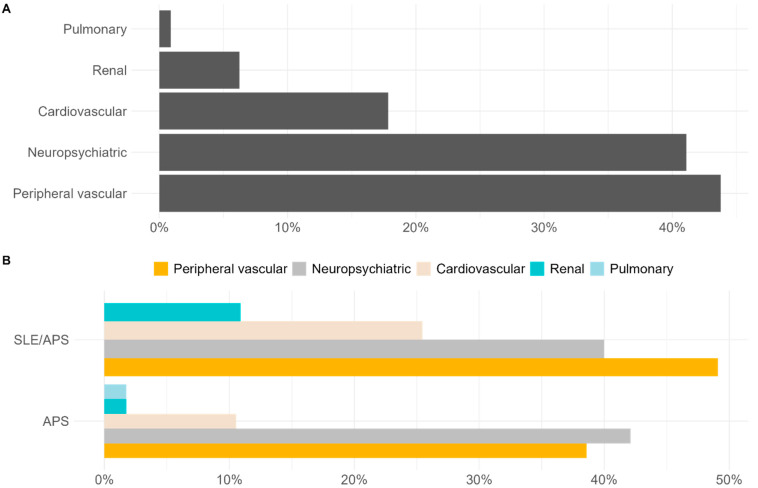

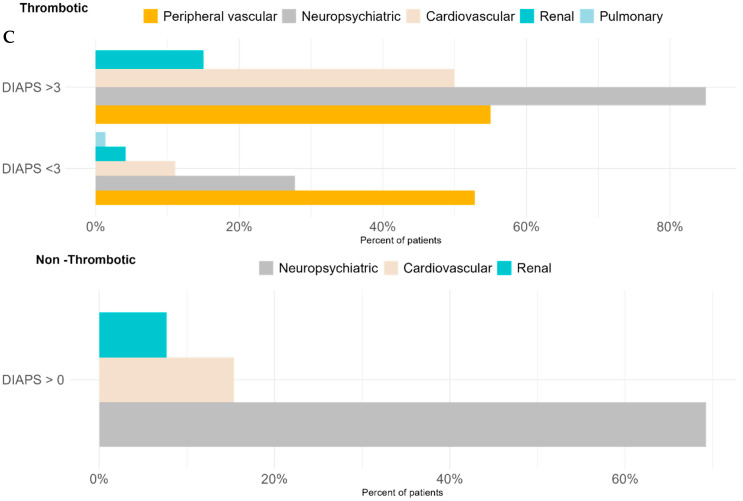
DIAPS domains overall (**A**), in APS and APS/SLE group (**B**) and in thrombotic and non-thrombotic group (**C**). Thrombotic; n = 92 (DIAPS > 3 n = 20, DIAPS < 3 n = 72); non-thrombotic; n = 20 (DIAPS = 0 n = 7, DIAPS > 0 n = 13); atherosclerotic plaque was present in 27 (24%) of the patients in this study with the same frequency in APS and APS/SLE groups (*p* = 0.5446) and was independent of DIAPS severity (Table 5).

The peripheral vascular domain was the most affected in thrombotic patients at 49 (53.2%) followed by the neuropsychiatric (37; 40.20%) and the cardiovascular (n = 18, 19.5%) domains. In non-thrombotic patients, the neuropsychiatric domain was followed by cardiovascular and renal (Table 4, Tables S1 and S2, Figure 1 and Figure S1). All domains were significantly more frequently affected in patients with high damage.

Analyzing the specific domains of DIAPS between thrombotic and non-thrombotic patients, we observed the domination of neuropsychiatric and cardiovascular symptoms in both groups but at a much lower frequency in the non-thrombotic group. There was no difference between primary APS patients and those with accompanying SLE.

**Figure 2:**
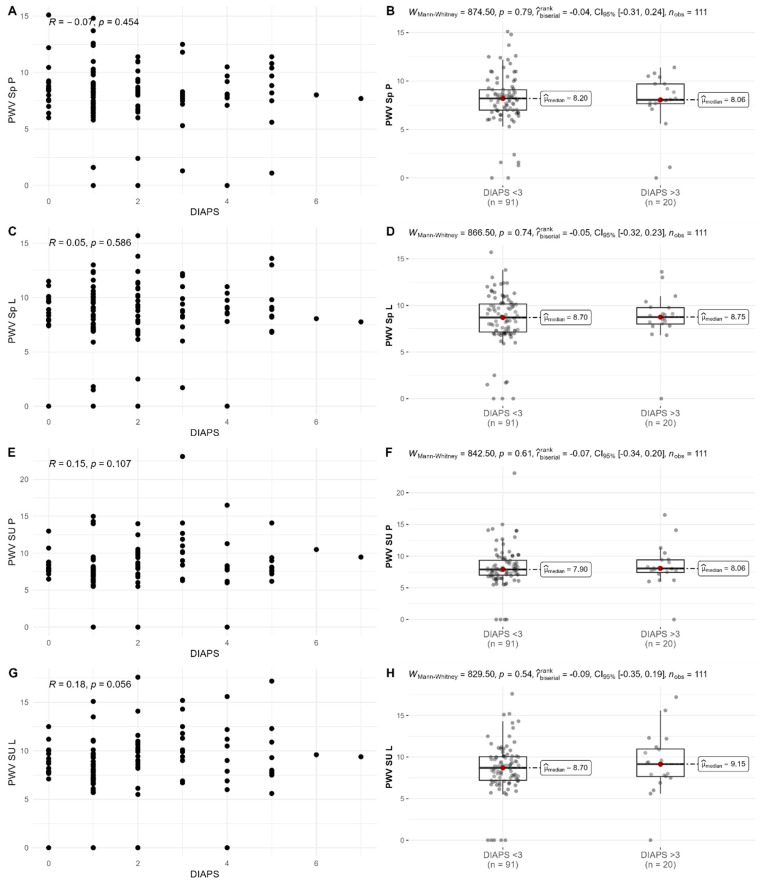
Pulse wave velocity (PWV) and damage (DIAPS). (**A**,**B**) PWV at right radial artery; (**C**,**D**) PWV at left radial artery; (**E**,**F**) PWV at right femoral artery; (**G**,**H**) PWV at left femoral artery.

Pulse wave velocity (PWV) was elevated in 27–32% of patients according to the analyzed arteries. Elevated PWV was more frequent in APS groups in comparison to APS/SLE, only between the carotid and radial arteries (*p* = 0.0012). PWV did not correlate with damage severity (Figure 2, Tables S3 and S4).

## 4. Discussion

The Damage Index for Antiphospholipid Syndrome (DIAPS) was developed to quantify irreversible organ damage in patients with antiphospholipid syndrome (APS) [11]. While it was initially designed for thrombotic APS, recent research shows that DIAPS can also be used to describe and assess damage in non-thrombotic patients with antiphospholipid antibodies (aPL) [13]. For the first time in a Polish population, we describe damage measured via DIAPS in APS patients. Damage was present in 86.6% of patients overall, including high (DIAPS > 3) in 17.86% in the thrombotic group. It corresponds with very similar results from the APS ACTION cohort (DIAPS in 85% of patients, with high damage in approximately 21%) [12]. Medina et al. found much higher rates of severe organ damage, 59.7% of thrombotic PAPS patients, with a median DIAPS value of 3 (IQR 2–5) [13]. These results are also discordant with previous reports from Erkan et al., where organ damage was observed in 38% of patients after 10 years of follow-up [14]; Grika et al. reported that 29% of 135 patients experienced damage assessed by SDI, after 7.5 years of follow-up [15]; or from Dall ‘Era et al., who described damage in 20% of 35 PAPS patients [16]. The was no significant discrepancy between studies for most affected domains of DIAPS in thrombotic patients [13,17,18,19]. Some heterogeneity may arise from differences between populations (with or without SLE, ethnicity, disease duration) and screening strategies. For example, patients with SLE and APS have a lower DIAPS at diagnosis but experience a greater and more persistent increase in damage over time, resulting in higher long-term DIAPS compared to patients without SLE, especially in those with neuropsychiatric and pulmonary involvement [17,19].

DIAPS is sensitive to a range of APS-related damage, including non-thrombotic manifestations such as neurological, cutaneous, and hematologic features, which are common in non-thrombotic APS patients [20]. Similarly to other publications [11], in a minority of APS patients, we observed non-thrombotic manifestations, and in most of them, DIAPS > 0 (65%). A notable proportion of non-thrombotic APS patients have measurable organ damage, as assessed by DIAPS, with cardiovascular risk factors and certain antibody profiles increasing the risk in the literature [12,17,20].

The important value of our study is the observation of subclinical atherosclerosis presenting data from Polish patients with APS for the first time. Risk factors for high damage in thrombotic patients were cardiovascular complications, especially myocardial infarction and reocclusion after CABG/PCI, as well as ASA therapy. At the same time, AP presence and increased PWV did not correlate with damage, which may also be partially due to the small sample size and inadequate statistical power. It is well known that cardiovascular complications and associated prophylaxis with ASA change the vessel wall, especially endothelium, which, in the presence of aPL, can trigger recurrent thrombosis and, in this way, increase DIAPS [21,22,23]. In the APS ACTION study, the presence of traditional CVD risk factors was associated with higher damage in both thrombotic and non-thrombotic aPL-positive patients, which we did not observe in our study. In a similar study to ours, including both SLE/APS and APS patients, Uludag et al. identified a cluster of patients with CVD risk factors and predominance of arterial events (n = 74) with a mean DIAPS of 2.24 (1.44) [18].

The arterial wall changes, especially in the endothelium, preceding CV clinical events and atherosclerosis. Subclinical atherosclerosis can be evaluated using ultrasonography imaging AP [24]. CA had a better predictive value than intima-media thickness alone for the risk of future cardiac and cerebral events [25]. Overall, in the literature published to date, in APS patients, AP was more prevalent than in controls [25]. Convincing data coming from Evangelatos et al. [26] showed higher AP presence in femoral arteries in APS patients with no traditional risk factors for atherosclerosis (e.g., age, dyslipidemia, and cumulative steroid dose). Interestingly, APS patients demonstrated a three-times larger risk of a new atherosclerotic plaque in this 3-year longitudinal study, which compared risk for atherosclerosis in patients with APS, diabetes mellitus or healthy controls [26]. In this study, plaque development risk was larger in the SLE/APS group versus patients with primary APS [26], contrary to our observations. However, in this study, the number of patients was lower, which may have influenced the results.

Measuring carotid plaque in patients with antiphospholipid syndrome (APS) is clinically relevant because it identifies a significantly increased risk of subclinical and progressive atherosclerosis, which is associated with higher rates of cardiovascular events. The clinical utility of AP measurement in APS includes the early identification of high-risk patients, guiding the intensity of cardiovascular prevention and monitoring disease progression and therapy response [10,27,28]. Carotid plaque assessment can help guide early intervention and more aggressive management of cardiovascular risk factors in APS patients. This approach can help reduce the long-term burden of arterial events in APS patients [29].

Arterial stiffness arises because of degeneration of the extracellular matrix in elastic arteries, mainly due to aging and the presence of CV risk factors. It has been hypothesized that increased stiffness and pulse pressure can lead to atherosclerosis [30]. Most frequently, it is measured using PWV [30]. PWV measurement was identified as a predictor of future CV events, where a 1 m/s increment in PWV resulted in a 7% increased risk of CV events [30]. PWV measurement in APS can identify early vascular dysfunction and help stratify cardiovascular risk, especially in patients with arterial events. While not universally required for all APS patients, it may be a valuable tool for targeted screening and management in higher-risk individuals [31].

We found that APS patients have increased PWV relative to the norm in the Polish population [32]. No difference was found between the APS and APS/SLE groups. In this study, evaluating PWV in APS, PWV values were also higher in APS patients than in healthy controls and comparable to patients with diabetes mellitus, used as a prototype for high CVD risk disorder [33]. Conversely, in the work of Andrade et al. [31], APS patients and controls had comparable PWV values in one case–control study; however, thrombotic patients with arterial events presented increased PWV compared to patients with venous thrombosis [31]. The small sample size (n = 27) is an important limitation of this observation. There was also one study that measured PWV between the carotid and femoral artery only in patients with no hypertension, which may be the reason for differences in the results compared to our study [28]. The measurement of PWV was comparable between the APS and APS/SLE groups in our analysis, except PWV between the carotid and radial arteries, which was increased in APS patients (46% vs. 17%, *p* = 0.0012) (Supplementary Materials). This needs to be further evaluated in future studies with bigger sample sizes, as such data do not exist in the public domain. According to our study, it seems that CVD complications’ history, but not AP presence or increased PWV, is a predictor of DIAPS in APS patients. Since data are scarce, there is a need for more studies with higher sample sizes to better understand the predictive value of arterial stiffness measurement. It may be important to focus on patients with no concomitant SLE and with no hypertension for PWV.

Another strength of this study is the wide panel of analyzed aPL. Contrary to other studies, we did not identify any aPL presence (single, dual or triple) associated with DIAPS. In the APS ACTION study, high titers of aβ_2_GPI correlated with high damage in thrombotic PAPS patients, and single aPL positivity negatively correlated with damage in the non-thrombotic group [9].

Our study also has limitations. It includes both PAPS and SLE-associated APS patients, which may introduce confounding factors. Patients with SLE may experience a greater and more persistent increase in damage over time, resulting in higher long-term DIAPS compared to patients without SLE [17]. While the limited sample size is significant for one center, it does not allow for the wide generalizability of our conclusions.

In conclusion, DIAPS indicates substantial damage in APS patients in our study. Almost one in five thrombotic APS patients presented with high organ damage, with the most frequently affected domains in thrombotic patients being peripheral vascular, neuropsychiatric and cardiovascular, as opposed to the neuropsychiatric and cardiovascular domains in non-thrombotic patients. CVD complications, myocardial infarct and reocclusion after CABG/PCI and ASA therapy correlated with higher damage in thrombotic primary APS patients, but AP presence and PWV did not. Prospective studies with a bigger patient sample are needed to better understand the damage accrual in APS patients with CVD risk factors.

High organ damage mainly affects thrombotic patients and is related to CVD complications. At the same time, the screening of subclinical atherosclerosis seems not to predict higher damage in APS patients.

## Figures and Tables

**Table 1:** Patients’ characteristics.

	Thrombotic (N = 92)				Non-Thrombotic (N = 20)				Thrombotic vs. Non-Thrombotic
	All (N = 92)	DIAPS < 3 (N = 72)	DIAPS > 3 (N = 20)	*p*-Value	All (N = 20)	DIAPS = 0 (N = 7)	DIAPS > 0 (N = 13)	*p*-Value	*p*-Value
Female, n (%)	78 (84.78%)	60 (83.33%)	18 (90.00%)	0.533	19 (95.00%)	7 (100.00%)	12 (92.31%)	1.000	0.307
Age, mean ± SD	48.49 ± 13.76	47.83 ± 14.41	50.85 ± 11.12	0.323	47.35 ± 13.67	41.71 ± 12.16	50.38 ± 13.91	0.171	0.738
Disease duration, median (1stQ, 3rdQ)	60.00 (24.00, 132.00)	60.00 (17.25, 123.00)	83.00 (30.25, 150.00)	0.218	99.00 (33.00, 315.00)	24.00 (12.00, 198.00)	144.00 (84.00, 300.00)	0.112	0.089
**Criteria Manifestation**									
Obstetric APS, n (%)	24 (26.09%)	17 (23.61%)	7 (35.00%)	0.460	15 (75.00%)	6 (85.71%)	9 (69.23%)	0.605	**<0.001**
Thrombosis overall, n (%)	92 (100.00%)	72 (100.00%)	20 (100.00%)	-	0 (0.00%)	0 (0.00%)	0 (0.00%)	-	-
Arterial thrombosis, n (%)	49 (53.26%)	34 (47.22%)	15 (75.00%)	0.051	0 (0.00%)	0 (0.00%)	0 (0.00%)	-	-
Venous thrombosis, n (%)	58 (63.04%)	46 (63.89%)	12 (60.00%)	0.955	0 (0.00%)	0 (0.00%)	0 (0.00%)	-	-
Patients at CVD risk, n (%) *	86 (93.48%)	67 (93.06%)	19 (95.00%)	1.00	16 (80.00%)	5 (71.43%)	11 (84.62%)	0.97	0.805
BMI, mean ± SD	25.95 ± 4.72	25.76 ± 4.89	26.64 ± 4.10	0.421	24.52 ± 4.38	22.69 ± 4.77	25.50 ± 4.01	0.213	0.201
Waist circumference, mean ± SD	78.23 ± 13.05	78.04 ± 12.71	78.90 ± 14.55	0.812	76.20 ± 11.69	71.29 ± 11.79	78.85 ± 11.19	0.189	0.496
hypertension, n (%)	36 (39.13%)	25 (34.72%)	11 (55.00%)	0.166	8 (40.00%)	2 (28.57%)	6 (46.15%)	0.646	1.000
Type 2 diabetes, n (%)	6 (6.52%)	5 (6.94%)	1 (5.00%)	1.000	2 (10.00%)	0 (0.00%)	2 (15.38%)	0.506	0.605
CVD complications, n (%)	45 (48.91%)	30 (41.67%)	15 (75.00%)	**0.017**	0 (0.00%)	0 (0.00%)	0 (0.00%)	**-**	**<0.001**
Stroke, n (%)	23 (25.00%)	16 (22.22%)	7 (35.00%)	0.199	0 (0.00%)	0 (0.00%)	0 (0.00%)	-	**0.052**
TIA, n (%)	18 (19.57%)	12 (16.67%)	6 (30.00%)	0.212	0 (0.00%)	0 (0.00%)	0 (0.00%)	-	**0.044**
Myocardial infarction, n (%)	10 (10.87%)	4 (5.56%)	6 (30.00%)	**0.007**	0 (0.00%)	0 (0.00%)	0 (0.00%)	-	0.196
Reoclusion after PCI/CABG, n (%)	3 (3.26%)	0 (0.00%)	3 (15.00%)	**0.011**	1 (5.00%)	0 (0.00%)	1 (7.69%)	1.000	1.000
**Antiphospholipid antibodies, n (%)**									
aCL IgG+, n (%)	18 (19.57%)	16 (22.22%)	2 (10.00%)	0.355	4 (20.00%)	0 (0.00%)	4 (30.77%)	0.265	1.000
aCL IgM+, n (%)	8 (8.70%)	7 (9.72%)	1 (5.00%)	0.681	3 (15.00%)	1 (14.29%)	2 (15.38%)	1.000	0.422
aβ2GPI IgG+, n (%)	61 (66.30%)	50 (69.44%)	11 (55.00%)	0.346	13 (65.00%)	3 (42.86%)	10 (76.92%)	0.182	1.000
aβ2GPI IgM+, n (%)	15 (16.30%)	11 (15.28%)	4 (20.00%)	0.717	5 (25.00%)	3 (42.86%)	2 (15.38%)	0.281	0.504
LAC+, n (%)	78 (84.78%)	62 (86.11%)	16 (80.00%)	0.731	15 (75.00%)	5 (71.43%)	10 (76.92%)	1.000	0.325
aCL, aß2GPI or LAC presence, n (%)	90 (97.83%)	70 (97.22%)	20 (100.00%)	1.000	20 (100.00%)	7 (100.00%)	13 (100.00%)	-	1.000
Double positive (aCL+ aβ2GPI, aβ2GPI + LAC, LAC + aCL)	46 (50.00%)	37 (51.39%)	9 (45.00%)	0.800	6 (30.00%)	2 (28.57%)	4 (30.77%)	1.000	0.141
Triple positive (aß2GPI + aCL + LAC)	16 (17.39%)	15 (20.83%)	1 (5.00%)	0.185	5 (25.00%)	1 (14.29%)	4 (30.77%)	0.624	0.545
**Hematological Manifestations**									
overall, n (%)	42 (45.65%)	34 (47.22%)	8 (40.00%)	0.749	10 (50.00%)	5 (71.43%)	5 (38.46%)	0.357	0.809
Leucopenia, n (%)	16 (17.39%)	14 (19.44%)	2 (10.00%)	0.515	6 (30.00%)	2 (28.57%)	4 (30.77%)	1.000	0.222
Anemia, n (%)	19 (20.65%)	14 (19.44%)	5 (25.00%)	0.762	4 (20.00%)	3 (42.86%)	1 (7.69%)	0.104	1.000
Thrombocytopenia, n (%)	18 (19.57%)	14 (19.44%)	4 (20.00%)	1.000	4 (20.00%)	1 (14.29%)	3 (23.08%)	1.000	1.000
Increased inflammatory parameters, n (%)	83 (90.22%)	66 (91.67%)	17 (85.00%)	0.394	19 (95.00%)	6 (85.71%)	13 (100.00%)	0.341	0.679
↑ ESR, n (%)	62 (67.39%)	47 (65.28%)	15 (75.00%)	0.582	15 (75.00%)	5 (71.43%)	10 (76.92%)	1.000	0.591
↑ CRP, n (%)	72 (78.26%)	58 (80.56%)	14 (70.00%)	0.354	12 (60.00%)	3 (42.86%)	9 (69.23%)	0.362	0.142
creatinine > 1.2 mg/dl, n (%)	13 (14.13%)	10 (13.89%)	3 (15.00%)	1.000	0 (0.00%)	0 (0.00%)	0 (0.00%)	1.000	0.124
GFR < 60 mL/min, n (%)	15 (16.30%)	8 (11.11%)	7 (35.00%)	0.015	0 (0.00%)	0 (0.00%)	0 (0.00%)	0.015	0.059
proteinuria, n (%)	12 (13.04%)	7 (9.72%)	5 (25.00%)	0.107	2/19 (10.53%)	0/6 (0.00%)	2 (15.38%)	0.534	1.000
low C3, n (%)	30 (32.61%)	25 (34.72%)	5 (25.00%)	0.582	6 (30.00%)	1 (14.29%)	5 (38.46%)	0.355	1.000
low C4, n (%)	15 (16.30%)	12 (16.67%)	3 (15.00%)	1.000	7 (35.00%)	2 (28.57%)	5 (38.46%)	1.000	0.083
**Immunosuppresive treatment**									
xucocorticoids, n (%)	46 (50.00%)	34 (47.22%)	12 (60.00%)	0.448	8 (40.00%)	3 (42.86%)	5 (38.46%)	1.000	0.488
mycophenolan mofetile, n (%)	4 (4.35%)	3 (4.17%)	1 (5.00%)	1.000	0 (0.00%)	0 (0.00%)	0 (0.00%)	1.000	0.584
cyclofosfamide, n (%)	12 (13.04%)	8 (11.11%)	4 (20.00%)	0.464	0 (0.00%)	0 (0.00%)	0 (0.00%)	0.464	0.129
azatioprine, n (%)	15 (16.30%)	12 (16.67%)	3 (15.00%)	1.000	0 (0.00%)	0 (0.00%)	0 (0.00%)	1.000	0.058
metotrexat, n (%)	13 (14.13%)	12 (16.67%)	1 (5.00%)	0.442	1 (5.00%)	1 (14.29%)	0 (0.00%)	0.338	0.457
belimumab, n (%)	1 (1.09%)	1 (1.39%)	0 (0.00%)	1.000	0 (0.00%)	0 (0.00%)	0 (0.00%)	1.000	1.000
**Anticoagulants**									
enoxaparine n (%)	6 (6.52%)	5 (6.94%)	1 (5.00%)	1.000	1 (5.00%)	1 (14.29%)	0 (0.00%)	0.360	1.000
acenokumarol, n (%)	35 (38.04%)	27 (37.50%)	8 (40.00%)	1.000	2 (10.00%)	1 (14.29%)	1 (7.69%)	1.000	0.018
warfarine, n (%)	23 (25.00%)	19 (26.39%)	4 (20.00%)	0.770	6 (30.00%)	2 (28.57%)	4 (30.77%)	1.000	0.769
acetylosalicic acid (ASA), n (%)	29 (31.52%)	16 (22.22%)	13 (65.00%)	<0.001	6 (30.00%)	1 (14.29%)	5 (38.46%)	0.345	1.000
clopidogrel, n (%)	2 (2.17%)	1 (1.39%)	1 (5.00%)	0.394	0 (0.00%)	0 (0.00%)	0 (0.00%)	0.394	1.000
riwaroksaban, n (%)	17 (18.48%)	14 (19.44%)	3 (15.00%)	0.768	2 (10.00%)	2 (28.57%)	0 (0.00%)	0.107	0.527
**Other treatment**									
statins, n (%)	10 (10.87%)	7 (9.72%)	3 (15.00%)	0.690	0 (0.00%)	0 (0.00%)	0 (0.00%)	0.690	0.196
chlorochine, n (%)	22 (23.91%)	15 (20.83%)	7 (35.00%)	0.229	2 (10.00%)	1 (14.29%)	1 (7.69%)	1.000	0.232
hydroxychlorochine, n (%)	47 (51.09%)	37 (51.39%)	10 (50.00%)	1.000	14 (70.00%)	5 (71.43%)	9 (69.23%)	1.000	0.145

* cardiovascular risk factors: hypertension, diabetes type 2, men over 55 years, women over 65 years, documented atherosclerosis complications—myocardial infarction, stroke, TIA, atherosclerotic occlusion of distal arteries, reocclusion after PCI/CABG, BMI > 30, HDL < 40 mg/dL, LDL > 100 mg/dL; ↑—increasedStatistical analyses were performed using the chi-square test for categorical variables, and *t*-test or Wilcoxon rank-sum test for continuous variables, depending on the normality of data distribution. When assumptions for the chi-square test were not met, *p*-values were simulated using Monte Carlo methods.

**Table 2:** Damage indices in the analyzed group. (**A**) DIAPS in SLE/APS and APS groups.

		All Patients (N = 112)			Non-Thrombotic (N = 20)		Thrombotic (N = 92)		*p*			
**DIAPS**												
DIAPS > 3, n (%)		20 (17.86%)			0 (0.00%)		20 (21.74%)					
DIAPS, median (1stQ, 3rdQ)		2.00 (1.00, 3.00)			1.00 (0.00, 2.00)		2.00 (1.00, 3.00)		<**0.001**			
		**SLE/APS**					**APS**					
		**non-thrombotic (N = 8)**			**thrombotic (N = 47)**		**non-thrombotic (N = 12)**			**thrombotic (N = 45)**		
**DIAPS > 3, n (%)**		0 (0.00%)			12 (25.53%)		0 (0.00%)			8 (17.78%)		
**DIAPS,** **median (1stQ, 3rdQ)**		1.00 (0.00, 1.00)			2.00 (1.00, 3.50)		1.00 (0.00, 2.00)			1.00 (1.00, 3.00)		
**(** **A** **)**												
	**Thrombotic** **(N = 92)**						**Non-Thrombotic** **(N = 20)**					
	**All**		**APS** **(N = 45)**	**TRU/APS** **(N = 47)**	***p***		**All**	**APS** **(N = 12)**			**TRU/APS** **(N = 8)**	***p***
**DIAPS** **>3, n (%)**	20 (21.74%)		8 (17.78%)	12 (25.53%)	ns	**DIAPS** **= 0, n (%)**	7 (35.00%)	4 (33.33%)			3 (37.50%)	ns
**DIAPS** **< 3, n (%)**	72 (78.26%)		37 (82.22%)	35 (74.47%)		**DIAPS** **> 0, n (%)**	13 (65.00%)	8 (66.67%)			5 (62.50%)	

**Table 3:** SLICC in the analyzed groups.

	All Patients (N = 112)		Non-Thrombotic (N = 20)		Thrombotic (N = 92)	*p* Value
**SLICC**						
SLICC > 3, n (%)	18 (16.07%)		0 (0.00%)		18 (19.57%)	
SLICC, median (1stQ, 3rdQ)	1.00 (1.00, 3.00)		1.00 (0.75, 2.00)		2.00 (1.00, 3.00)	0.044
	**SLE/APS**			**APS**		
	All (N = 55)	non-thrombotic (N = 8)	Thrombotic (N = 47)	All (N = 57)	non-thrombotic (N = 12)	Thrombotic (N = 45)
**SLICC**						
SLICC > 3, n (%)	12 (21.82%)	0 (0.00%)	12 (25.53%)	6 (10.53%)	0 (0.00%)	6 (13.33%)
SLICC, median (1stQ, 3rdQ)	2.00 (1.00, 3.00)	1.00 (0.75, 1.00)	2.00 (1.00, 3.50)	1.00 (1.00, 2.00)	1.00 (0.75, 2.00)	1.00 (1.00, 2.00)

**Table 4:** DIAPS domains: thrombotic and non-thrombotic groups.

	Thrombotic		Non-Thrombotic	
	DIAPS < 3 (N = 72)	DIAPS > 3 (N = 20)	DIAPS = 0 (N = 7)	DIAPS > 0 (N = 13)
**DIAPS domain**				
Peripheral vascular	38 (52.78%)	11 (55.00%)	0 (0.00%)	0 (0.00%)
Pulmonary	1 (1.39%)	0 (0.00%)	0 (0.00%)	0 (0.00%)
Gastrointestinal	0 (0.00%)	0 (0.00%)	0 (0.00%)	0 (0.00%)
Renal	3 (4.17%)	3 (15.00%)	0 (0.00%)	1 (7.69%)
Cardiovascular	8 (11.11%)	10 (50.00%)	0 (0.00%)	2 (15.38%)
Neuropsychiatric	20 (27.78%)	17 (85.00%)	0 (0.00%)	9 (69.23%)
Musculoskeletal	0 (0.00%)	1 (5.00%)	0 (0.00%)	0 (0.00%)
Cutaneous	0 (0.00%)	1 (5.00%)	0 (0.00%)	0 (0.00%)
Ophthalmologic	0 (0.00%)	1 (5.00%)	0 (0.00%)	0 (0.00%)
Endocrine	0 (0.00%)	0 (0.00%)	0 (0.00%)	0 (0.00%)

**Table 5:** Presence of atherosclerotic plaque (AP) and damage according to DIAPS.

	**AP Presence in Carotid Arteries**		
	No (N = 84)	yes (N = 27)	*p*
DIAPS > 3, n (%)	15 (17.86%)	5 (18.52%)	ns
DIAPS, median (1stQ, 3rdQ)	1.00 (1.00, 3.00)	2.00 (1.00, 3.00)	ns
	**AP Presence in the Right Carotid Artery**		
	No (N = 88)	Yes (N = 23)	
DIAPS > 3, n (%)	15 (17.05%)	5 (21.74%)	ns
DIAPS, median (1stQ, 3rdQ)	1.50 (1.00, 3.00)	2.00 (1.00, 3.00)	ns
	**AP Presence in the Left Carotid Artery**		
	No (N = 90)	Yes (N = 21)	
DIAPS > 3, n (%)	16 (17.78%)	4 (19.05%)	ns
DIAPS, median (1stQ, 3rdQ)	1.00 (1.00, 3.00)	2.00 (1.00, 3.00)	ns

## Data Availability

Data supporting the reported results can be found at NIGRIR.

## Supplementary Materials

The following Supporting Information can be downloaded at: https://www.mdpi.com/article/10.3390/biomedicines13071671/s1, Figure S1: DIAPS and SLICC in APS and ASP/SLE groups; Table S1: DIAPS in SLE/APS and APS groups; Table S2: Detailed DIAPS domains: thrombotic and non-thrombotic groups; Table S3: AP and PWV in the whole group; Table S4: AP and PWV in the APS and APS/SLE subgroups.
