# Supplementary material for: Damage Burden in Polish Patients with Antiphospholipid Syndrome Measured Using Damage Index for Antiphospholipid Syndrome (DIAPS)
Source: Biomedicines. 2025 Jul 8;13(7):1671. doi: 10.3390/biomedicines13071671 (PMC12292454; doi:10.3390/biomedicines13071671)
Supplement: Supplementary file 1 [file biomedicines-13-01671-s001.zip › biomedicines-3633237-supplementary.pdf]

Figure S1 (supplement) DIAPS and SLICC in APS and ASP/SLE groups.

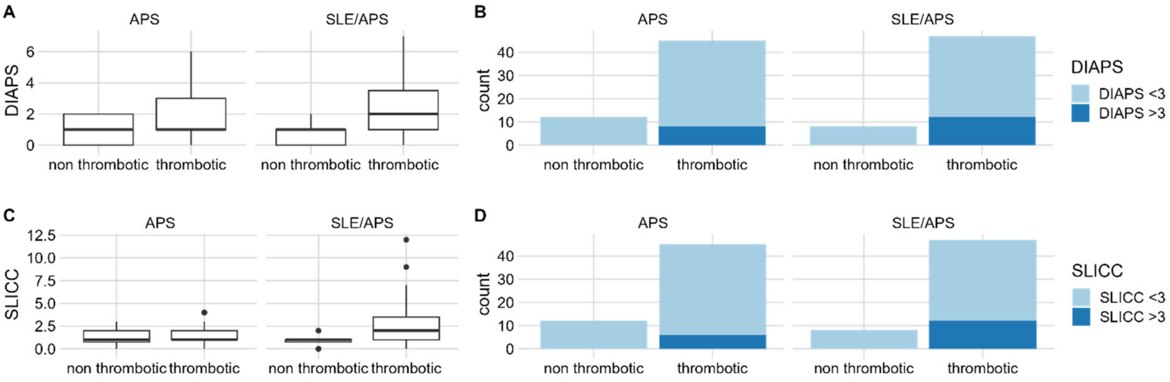

Table S1 (supplement) DIAPS in SLE/APS and APS groups.

	thrombotic (N = 92)					Non thrombotic (N = 20)			
** **	All	APS (N = 45)	TRU/APS (N = 47)	p	** **	All	APS (N = 12)	TRU/APS (N = 8)	p
DIAPS >3, n (%)	20 (21.74%)	8 (17.78%)	12 (25.53%)	ns	DIAPS =0, n (%)	7 (35.00%)	4 (33.33%)	3 (37.50%)	ns
DIAPS <3, n (%)	72 (78.26%)	37 (82.22%)	35 (74.47%)		DIAPS >0, n (%)	13 (65.00%)	8 (66.67%)	5 (62.50%)	

Table S2. Detailed DIAPS domains thrombotic and non-thrombotic groups.

	All patients		APS		SLE/APS	
	non thrombotic (N = 20)	thrombotic (N = 92)	non thrombotic (N = 12)	thrombotic (N = 45)	non thrombotic (N = 8)	thrombotic (N = 47)
**Peripheral vascular**						
DVT, n (%)	0 (0.00%)	44 (47.83%)	0 (0.00%)	21 (46.67%)	0 (0.00%)	23 (48.94%)
Tissue loss: minor, n (%)	0 (0.00%)	9 (9.78%)	0 (0.00%)	0 (0.00%)	0 (0.00%)	9 (19.15%)
Tissue loss: major, n (%)	0 (0.00%)	3 (3.26%)	0 (0.00%)	1 (2.22%)	0 (0.00%)	2 (4.26%)
Vascular venous insufficiency, n (%)	0 (0.00%)	11 (11.96%)	0 (0.00%)	2 (4.44%)	0 (0.00%)	9 (19.15%)

Pulmonary						
Pulmonary infarction, n (%)	0 (0.00%)	0 (0.00%)	0 (0.00%)	0 (0.00%)	0 (0.00%)	0 (0.00%)
Pulmonary arterial hypertension (%)	0 (0.00%)	1 (1.09%)	0 (0.00%)	1 (2.22%)	0 (0.00%)	0 (0.00%)
Chronic thromboembolic pulmonary hypertension, n (%)	0 (0.00%)	0 (0.00%)	0 (0.00%)	0 (0.00%)	0 (0.00%)	0 (0.00%)
Respiratory insufficiency, n (%)	0 (0.00%)	0 (0.00%)	0 (0.00%)	0 (0.00%)	0 (0.00%)	0 (0.00%)
Gastrointestinal						
Mesenteric thrombosis, n (%)	0 (0.00%)	0 (0.00%)	0 (0.00%)	0 (0.00%)	0 (0.00%)	0 (0.00%)
Budd-Chiari syndrome, n (%)	0 (0.00%)	0 (0.00%)	0 (0.00%)	0 (0.00%)	0 (0.00%)	0 (0.00%)
liver cirrosis, n (%)	0 (0.00%)	0 (0.00%)	0 (0.00%)	0 (0.00%)	0 (0.00%)	0 (0.00%)
Renal						
thrombotic renal microangiopathy, n (%)	1 (5.00%)	1 (1.09%)	1 (8.33%)	0 (0.00%)	0 (0.00%)	1 (2.13%)
Chronic renal failure, n (%)	0 (0.00%)	5 (5.43%)	0 (0.00%)	0 (0.00%)	0 (0.00%)	5 (10.64%)
proteinuria>3,5g/24 h , n (%)	0 (0.00%)	2 (2.17%)	0 (0.00%)	0 (0.00%)	0 (0.00%)	2 (4.26%)
Cardiovascular						
Coronary artery bypass, n (%)	0 (0.00%)	1 (1.09%)	0 (0.00%)	0 (0.00%)	0 (0.00%)	1 (2.13%)
myocardial infarction, n (%)	0 (0.00%)	11 (11.96%)	0 (0.00%)	4 (8.89%)	0 (0.00%)	7 (14.89%)
cardiomiopathy, n (%)	1 (5.00%)	3 (3.26%)	0 (0.00%)	2 (4.44%)	1 (12.50%)	1 (2.13%)
aPL-associated heart valve disease, n (%)	1 (5.00%)	6 (6.52%)	0 (0.00%)	1 (2.22%)	1 (12.50%)	5 (10.64%)
aPL-associated heart valve disease with replacement, n (%)	0 (0.00%)	1 (1.09%)	0 (0.00%)	0 (0.00%)	0 (0.00%)	1 (2.13%)

Neuropsychiatric						
Cognitive impairment, n (%)	3 (15.00%)	20 (21.74%)	3 (25.00%)	8 (17.78%)	0 (0.00%)	12 (25.53%)
seizures, n (%)	4 (20.00%)	13 (14.13%)	2 (16.67%)	5 (11.11%)	2 (25.00%)	8 (17.02%)
Ischemic stroke with hemiparesia, n (%)	0 (0.00%)	12 (13.04%)	0 (0.00%)	4 (8.89%)	0 (0.00%)	8 (17.02%)
Ischemic stroke with hemiplegia, n (%)	0 (0.00%)	6 (6.52%)	0 (0.00%)	5 (11.11%)	0 (0.00%)	1 (2.13%)
Peripheral neuropathy, n (%)	2 (10.00%)	3 (3.26%)	2 (16.67%)	2 (4.44%)	0 (0.00%)	1 (2.13%)
Multi-infarct dementia, n (%)	1 (5.00%)	9 (9.78%)	1 (8.33%)	3 (6.67%)	0 (0.00%)	6 (12.77%)
Optic neuropathy, n (%)	0 (0.00%)	2 (2.17%)	0 (0.00%)	1 (2.22%)	0 (0.00%)	1 (2.13%)
Sudden sensorineural hearing loss, n (%)	0 (0.00%)	0 (0.00%)	0 (0.00%)	0 (0.00%)	0 (0.00%)	0 (0.00%)
Cranial neuropathy, n (%)	0 (0.00%)	0 (0.00%)	0 (0.00%)	0 (0.00%)	0 (0.00%)	0 (0.00%)
Abnormal movements, n (%)	0 (0.00%)	0 (0.00%)	0 (0.00%)	0 (0.00%)	0 (0.00%)	0 (0.00%)
Musculoskeletal						
avascular necrosis, n (%)	0 (0.00%)	1 (1.09%)	0 (0.00%)	0 (0.00%)	0 (0.00%)	1 (2.13%)
Cutaneous						
chronic cutaneous ulcersa, n (%)	0 (0.00%)	1 (1.09%)	0 (0.00%)	0 (0.00%)	0 (0.00%)	1 (2.13%)
Ophthalmologic						
Retinal vaso-occlusive disease, n (%)	0 (0.00%)	0 (0.00%)	0 (0.00%)	0 (0.00%)	0 (0.00%)	0 (0.00%)
blindness, n (%)	0 (0.00%)	1 (1.09%)	0 (0.00%)	0 (0.00%)	0 (0.00%)	1 (2.13%)
Endocrine						
infertility, n (%)	0 (0.00%)	0 (0.00%)	0 (0.00%)	0 (0.00%)	0 (0.00%)	0 (0.00%)

Suprarenal insufficiency, n (%)	0 (0.00%)	0 (0.00%)	0 (0.00%)	0 (0.00%)	0 (0.00%)	0 (0.00%)
Hypopituitarismn (%)	0 (0.00%)	0 (0.00%)	0 (0.00%)	0 (0.00%)	0 (0.00%)	0 (0.00%)

Table S3. AP and PWV in the studied whole group.

APS+APS/SLE	Media ± SD<br>mediana (min.-max.)	N patients (%)
AP (mm)	2,22 ± 0,63	26 (23,4%)
PWV carotid-radial (m/s)	8.5± 2.19<br>8,5 (1,5-13,55)	35 (32,4%)
PWV carotid-femoral (m/s)	8.9± 2.26<br>8,3 (5,5-16,05)	29 (27,1%)

Table S4. AP and PWV in the APS i APS/SLE subgroups.

	APS		APS/SLE		P
	media ± SD<br>mediana (min.-max.)	N, %	media ± SD<br>mediana (min.-max.)	N, %	
AP (mm)	2,23± 0,63	12/57<br>(21,1%)	2,22 ± 0,73	14/55<br>(25,9%)	0,5446
PWV carotid-radial (m/s)	9.02± 2.3<br>9,02 (1,5-13,5)	26/56<br>(46,4%)	8.07± 1.98<br>8,25 (1,5-12)	9/52<br>(17,3%)	0,0012
PWV carotid-femoral (m/s)	8.88±2.57<br>8,05 (5,6-16)	17/56<br>(30,4%)	8.93±1,9<br>8,6 (5,5-15,65)	12/51<br>(23,5%)	0,4274
